# HF ultrasound *vs* PET-CT and telethermography in the diagnosis of In-transit metastases from melanoma: a prospective study and review of the literature

**DOI:** 10.1186/s13046-014-0096-3

**Published:** 2014-11-25

**Authors:** Francesco Maria Solivetti, Flora Desiderio, Antonino Guerrisi, Antonio Bonadies, Carlo Ludovico Maini, Simona Di Filippo, Valerio D’Orazi, Isabella Sperduti, Aldo Di Carlo

**Affiliations:** Radiology and Diagnostic Imaging Unit, IFO-San Gallicano Institute, Via Elio Chianesi 56, Rome, Italy; Plastic Surgery Department, IFO-San Gallicano Institute ISG, Via Elio Chianesi 56, Rome, Italy; Nuclear Medicine Unit, IFO-Regina Elena National Cancer Institute, Via Elio Chianesi 56, Rome, Italy; Surgery Department, Unit C IFO-Regina Elena National Cancer Institute, Via Elio Chianesi 56, Rome, Italy; Department of Surgical Sciences, Sapienza University, Rome, 00161 Italy; Biostatistical Unit, IFO-Regina Elena National Cancer Institute, Rome, Italy; IFO-San Gallicano Institute, Via Elio Chianesi 56, Rome, Italy

**Keywords:** High frequency-ultrasound, 18F-FDG PET-CT, Telethermography, Cutaneous melanoma, In transit-metastases, Electrochemotherapy

## Abstract

**Background:**

Over the past several years the incidence of cutaneous melanoma has rapidly increased. This tumor develops often in-transit metastases that significantly reduce patient survival at 5 years. To improve prognosis and quality of life in patients with melanoma metastases, a mini invasive procedure like electrochemotherapy (ECT) is adopted to remove superficial tissue lesions. To detect the melanoma metastases, high frequency (HF) ultrasound (US) is used. This technique, though, can be time-consuming and it needs an expert operator and a high performing machine. Therefore, we asked whether the US could be replaced or integrated with other less time-consuming techniques such as 18-FDG positron emission tomography/computed tomography (PET-CT) and telethermography (TT).

**Methods:**

Fifteen patients (4 males and 11 females - age range: 63–91) affected whit advanced stage melanoma were enrolled. They presented 52 in-transit metastases as detected by the three techniques used, HF-US, PET/CT and TT within 30 days before ECT.

**Results:**

All the 52 lesions were detected by HF-US (100%), 24/52 were detected by PET-CT (42,6%) and 15/52 were detected by TT (27,7%). PET-CT reported 3.7% false positives, while no false positive were reported by TT.

**Conclusions:**

As US detected 100% lesions, compared to the other two techniques used, US, along with clinical examination, has still to be considered as gold standard in the diagnosis of metastatic lesions. US, associated with an exhaustive anamnesis and accurate clinical examination, cannot be replaced by either PET-CT or TT. When US performing devices and experienced operators are not available, though, it is highly recommended to integrate US with at least one of the other techniques. Under certain circumstances, as in the case of obese and non-collaborating patients or in patients with lymphatic stasis, these techniques should be integrated to obtain exact in-transit metastases evaluation.

## Introduction

Over the past several years the incidence of cutaneous melanoma has rapidly increased (incidence ranging from 2 to 20%) with about 60,000 new cases diagnosed every year in Europe [1-3]. Five-year survival rate varies in a wide range (10-70%, depending on many variables) and it is reported to be much lower in patients with cutaneous metastases [4-8]. Cutaneous metastases from melanoma are very frequent and related to different risk factors and clinical parameters of which the most relevant are high thickness and number of mitoses, all determining tumor stage and consequently different patient management [9,10]. Clinically, these lesions could appear as solitary or multiple and present a papule-like or nodule-like morphology with possible ulceration as well as erysipeloid or erythematous patches but also widespread sclerodermiform shape (‘en cuirasse’) [2]. To improve prognosis and quality of life in patients with melanoma metastases, mini invasive procedure like electrochemotherapy (ECT) [11,12] is adopted to remove superficial tissue lesions. However, to choose the correct therapeutical approach, an exact localization of site, number and size of metastases as well as their depth are necessary [12-15]. To this purpose, at present, ultrasound (US) examination with high frequency (HF) [16,17] probes is considered to be the best diagnostic choice due to its high accuracy in detecting this kind of lesions. However, US has important limitations, such as prolonged execution time (not less than 30′-40′ for each limb or body area) and being much dependent on the operator’s skills, and the availability of a performing machine operated by an expert radiologist. Since we don’t have at the moment the possibility to perform in the normal clinical practice worldwide these specialized exams with the same protocol, an expert operator and US machine, we have probably to find a compromise among an acceptable sensitivity, the time needed to perform the exams and the real cost of these exams for the healthcare system. Therefore, it is important to evaluate whether other available techniques might have good sensitivity for detecting melanoma metastases in patients eligible for ECT, in order to replace or integrate US with other less time-consuming imaging techniques. In this respect, positron emission tomography/computed tomography (PET-CT) and telethermography (TT) can be considered useful to overcome these limitations and to obtain adequate diagnostic results. Therefore, the aim of this study was to compare US with 18-FDG PET-CT and TT in the detection of melanoma metastases in a subgroup of patients with advanced stage melanoma eligible for ECT.

## Materials and methods

### Ethics statement

This prospective single-centre, open-label study was approved by IFO-San Gallicano Institute review board and followed the principles of the Declaration of Helsinki and subsequent amendments. All participants provided written informed consent before participating in the study.

### Study population

We analyzed 52 lesions from 15 melanoma patients (age ranging from 63 to 91 years) scheduled for ECT treatment in the San Gallicano Institute, with HF-US, 18-FDG PET-CT, and TT. The whole evaluation was completed in a maximum of 30 days. All patients were followed up, as envisaged by standard protocols for melanoma, for at least one year. Inclusion criteria were anamnestic presence of melanoma and suspicious in-transit metastases. Before ECT, a microhistologic or cytological examination was performed on at least one of the nodules to be treated; diagnostic suspicion in the patients of this cohort was always confirmed. All patients underwent to PET/CT also for disease staging. Operators were blinded as for other examinations.

### High-frequency ultrasound (UF-US)

All HF-US examinations in our series were performed with an Esaote MyLab 70 XVG (Genoa, Italy), using a 18 MHz linear probe, at times integrated by a 13 MHz with specific setting. In selected cases, for very small and superficial lesions, an Esaote MyLab One with 22 MHz linear probe was utilized. Colour-Doppler US was performed with multiple samplings from different areas of the lesion thus allowing assessment of slow-flow conditions. The following lesions characteristics were reported: size, echogenicity, structure, margins, possible internal necrosis and their depth, besides vascularization pattern (1 = no vascularization, 2 = perilesional vascularization and 3 = intralesional vascularization). On the basis of these parameters, an overall numerical value was attributed to suspicious lesions between 0 and 3 (0 = benign lesion, 1 = possibly benign lesion, 2 = probably malignant lesion and 3 = malignant lesion) [18-20].

### Telethermography (TT)

Telethermography (TT) is a fast, unexpensive, non-invasive, and reproducible technique, which enables to transform into video images the infrared emissions of the human body on the basis of any possible changes of cutaneous temperature [21]. TT imaging gives the possibility to visualize, in an homogenous hypothermic domain, the presence of hot spots suggestive of cutaneous tumors [21-25]; in particular, this comes true in the presence of a very high hyperthermic gradient, at times with a specific pattern, as it is for melanoma (also known as “thermal flame”). For this particular examination we utilized FLIR 3000 Thermocam™ (Flir Systems Inc., Wilsonville, Or; USA) where the high spatial resolution of this infrared camera, associated with thermostimulation. This method utilizes a thermostimulator enabling to standardize contact times and thermostimulating temperatures. This makes it possible to detect very low thermal gradients, sometimes <0,01°C, increasing TT sensitivity, also in the case of low thermal gradients, as for small lesions (<0.5 cm), with consequent low emittance from the little tumor mass [21-25].

### Positron emission tomography/computed tomography (PET/CT)

PET/CT analyses were performed with a Biograph Sensation 16 (Siemens Medical Solutions, Erlangen, Germany). Following i.v. administration of specific drug such as 18-fluoro-2-deoxy-D-glucose (18-FDG) (Iason GmbH, Graz, Austria) at 10–15 mCi, diluted in 2–3 ml volume, the PET-CT protocol envisages caudo-cranial imaging through low-dose CT (120–140 kV); for mAs setting care-dose software is utilized, for optimization of supplied dose. PET examination is carried out from cranium to feet. In a whole-body PET an average of 15 visual fields is obtained, with image acquisition time of 2–3 minutes each. Coronal, sagittal and transversal images are acquired with TrueX reconstruction algorithm, 70 cm visual field and image matrix dimensions 128 × 128. CT images are reconstructed at a 512 × 512 pixel resolution while PET images with 168 × 168 pixel matrix with final fusion of the images.

### Electrochemotherapy (ECT)

Electrochemotherapy (ECT) utilizes high-frequency pulsed electricity to increase membrane permeability of neoplastic cells, thus creating micropores through which small drug molecules can spread throughout the cells. Bleomycin and cisplatin are the two more frequently utilized drugs [11]. The elevated in-cell drug concentration obtained with this technique, enables to improve the chemiotherapeutical efficacy of the agent which exerts its action locally at the electropermeabilization site.

### Statistical analyses

Descriptive statistics were computed for all variables. Continuous data were reported as the mean and categorical data were represented by frequencies and percentage values. Sensitivity (with the 95% confidence interval – CI -) was estimated considering the US results as the gold standard.

## Results

In our series, a total of 52 lesions were detected in 15 patients, as summarized in Table 1. Melanoma size varied between 1.2 and 2.8 mm, while lesions’ size was between 1.7 and 25 mm. US enabled to detect 52 lesions (size 1.7-25 mm, mean 7,1 mm; depth to epidermis 0–25 mm, mean 6.5 mm); PET-CT detected 24 lesions (size 4–25 mm, mean 10.8 mm; depth to epidermis 0–18 mm, mean 5.8 mm - data confirmed by US -); TT identified 15 lesions (size 3.7-25 mm, mean 12.8 mm; depth to epidermis 0–18 mm, mean 4.7 mm- data confirmed by US -) (Table 2). We found that 32 out of a total of 52 lesions (61.5%) were visualized only by the instruments since they were not clearly evident on clinical examination. US detected all 52 lesions with 100% sensitivity; PET-CT detected 24 lesions, with 46.2% (CI 95% 27.7/64.6) sensitivity; PET-CT had 2 false positive (3.7% of the total if related to the cohort in its entirety and 8.3% when referred to the 24 patients diagnosed with this technique); TT detected 15 lesions, with 28.8% (CI 95% 14.3/43.4) sensitivity, with no false positive results reported (Table 2). As expected, sensitivity results of PET-CT and TT varied according to lesions’ size, compared to US that reported 100% sensitivity at the smallest lesion’ size (Table 3).

**Table 1:** **Patients, melanoma and lesion characteristics**

**No.**	**Sex**	**Age (years)**	**Year of diagnosis**	**Melanoma thickness (mm)**	**Lesions’ size (mm)**	**Lesions’ depth (mm)**	**Lesions’ site**
1	M	80	2008	1.7	16	1.4	TRUNK
					4	4	TRUNK
2	F	64	2010	1.7	15	7	LEG SX
					25	2.5	LEG SX
3	F	71	2009	1.7	NEG	NEG	NEG
					10	10	INGUINALE
4	F	75	2008	2.4	20	18	POPLITEAL
5	F	67	2009	1.2	4	7	POPLITEAL
					NEG	NEG	NEG
6	F	65	2008	2.8	6	18	FOOT
					4	9	FOOT
					6	3	FOOT
7	M	77	1999	1.7	10	0	LEG
					3.7	3	LEG
					4.2	1	LEG
8	F	63	2000	1.7	4.3	8.6	FOREARM
					2.2	5	FOREARM
					1.9	4	FOREARM
					4.6	3	FOREARM
					4.6	3	FOREARM
					3	2	FOREARM
					5	3	FOREARM
					6	3	FOREARM
9	F	68	2009	1.7	9	2	FOREARM
					3	10	LEFT LEG
					3.2	4.5	LEFT LEG
					4.2	8	LEFT LEG
					4	3.5	LEFT LEG
					4	2.5	LEFT LEG
					4.6	1.5	LEFT LEG
					2	1.6	LEFT LEG
					4.3	25	LEFT LEG
10	M	80	2008	1.7	18	7	TRUNK
					23	5	TRUNK
11	F	78	2010		11	3	RIGHT LEG
					9	2	RIGHT LEG
12	F	91	20111		4	16	RIGHT LEG
13	F	83	2010		12	12	POPLITEAL CAVITY
					5	5	RIGHT LEG
					17	5	EXTERNAL MALLEOLUS
					14	12	LEG
14	M	75	2010		6	7	LEFT LEG
					4	4	LEFT LEG
					2.5	3	LEFT THIGH
					2.3	4	LEFT THIGH
					2	4	LEFT THIGH
					4	5	LEFT THIGH
					3	7	LEFT THIGH
					1.7	4	LEFT THIGH
					3	5	LEFT THIGH
					4.6	6	LEFT THIGH
					2	3	LEFT THIGH
15	F		2011		7	4	LEFT MEDIAL MALLEULUS
					4	5	LEFT LEG

**Table 2:** **Lesions detected by HF-US, PET/CT, and TT**

	**No lesions**	**Size (mm)**	**Depth to epidermis (mm)**	**Sensitivity (%)**
HF-US	52/52	1.7/25	0.25	100
PET-CT	24/52	4-25	0-18	46.2
TT	15/52	3.7-25	0-18	28.8

**Table 3:** **Sensitivity values for PET-CT and TT according to the lesions’ size; the sensitivity of US was 100%**

**Lesions**	**PET-CT**			**TT**		
Size (mm)	<4	5-6	>7	<4	5-7	>7
Sensitivity (SD)	26%	29%	93%	9%	15%	73%
	(5–47)	(1–56)	(44–100)	(1–21)	(1–37)	(30–100)

The vascularized lesions visualized by Doppler were 13 out of 15 melanomas (25%), larger than 7 mm and detectable at PET-CT examination. In particular, TT showed elevated sensitivity, 73.3% (CI 95% 30–100) for lesions >7 mm and located at a relatively limited distance from the cutaneous plane; such sensitivity increased with criostimulus as demonstrated by the fact that out of the 15 lesions detected by TT only 4 were <7 mm and all were superficial, located at a depth <7 mm from the cutaneous plane.

ECT was well tolerated by the great majority of patients enrolled in the study and the erithematous reaction, frequently associated to treatment, usually vanished in a few days even though it could sometimes form a sort of “scab” on treated areas [12,15]. Involuntary muscle contractions were reported in only 15% of patients recruited in this study. The lesions treated with ECT showed very little changes in the first 48 hours, while in the following few weeks slowly became less well contoured and hypoecogenic and with a reduction of their volume. The subcutaneous adipose tissue became instead more inhomogeneous, while superficial planes thickened and showed the tendency to inhomogeneous fibrous tissue changes.

## Discussion

The choice of a correct therapeutical approach for melanoma needs an exact localization of site, number and size of metastases as well as their depth [12-15]. HF-US is confirmed to be the best imaging technique available in the detection of melanoma in-transit-metastases [18,21,25-31]. However, given some reported limitations for HF-US, we wanted to compare HF-US technique with PET-CT and TT, which still present good sensitivity for detecting melanoma metastases in patients eligible for ECT. The purpose was to evaluate whether it was possible to replace or integrate HF-US with other less time-consuming imaging techniques. We found that US detected 100% of the in-transit metastases, compared to the other two techniques utilized, confirming the greater sensitivity of US in the diagnosis of subcutaneous lesions (nodes and satellitosis).

In this study, in-transit metastases appeared as subcutaneous nodules, hypoechoic often with very low internal echoes, because of poor melanin reflexion; margins were not seldom irregular or polycyclic. The largest lesions showed necrotic areas with anechoic appearance, however due to their dimensions (usually they are <1 cm) nodules appeared to be relatively homogeneous. Metastases were single or multiple within a tumor, often located between the primary lesion and the nodal basin draining that area. The US, associated with clinical examination, has generally considered the diagnostic gold standard for melanoma. In a relatively recent study that enrolled 600 patients with melanoma of clinically relevant thickness (>1 mm), but negative to objective examination at clinical follow-up, suspicious in-transit metastases were reported in 63 patients for a total amount of 95 lesions, all detected by US that did not report false positive or false negative results [18]. In addition, a recent study utilizing TT in the diagnosis of cutaneous metastases from melanoma on 74 patients presenting 251 lesions showed 95% sensitivity and 100% specificity for nodules >15 mm, and 58% sensitivity and 98% specificity for lesions between 5 and 15 mm [21]. These results though were less significant when the lesions extended to the deepest strata of teguments beyond 1.5 cm from epidermis [21], pointing at the importance of the lesions’ size for the sensitivity of techniques different than US. In agreement, here we confirmed the limit of TT in detecting small and/or deeply located metastatic lesions.

In our study, lesions that exhibited internal vascular signals at Doppler imaging were only 13 (24%); except for 4 lesions that presented minimal vascularization, the remnant 9 were >7 mm in diameter and all detected by PET-CT. In the literature, vascular signal at Doppler are reported to be detected in up to 70% of metastatic lesions, in particular the largest ones: such discrepancy can be easily explained by the short mean diameter of the lesions studied in our series (7 mm). Only 15 lesions were detected with TT, with a 28.8% sensitivity.

Concerning PET-CT, our results seem to confirm their validity only for vascularized and biologically active lesions, which usually correspond to high standard uptake value (SUV). This technique is anyhow limited in differential diagnosis with venous ectasia. Moreover SUV values do not seem to be directly linked to clinical significance of the analysed sample, being then not always specific. When carried out routinely, PET-CT does not allow to include the interested area, i.e. the hand, and therefore sometimes need a second scansion, with different protocols, thus creating further delay, cost rising, and limited availability, despite the general value of PET-CT in tumor staging cannot be denied. If more than one lesion of the surrounding tissue is detected, only seldom is possible to detect their exact number since they form a unique capturing mass. PET-CT has not a high spatial resolution and, as confirmed in our series, the minimal detectable dimension of the lesion must be at least 5 mm [30], as demostrated also by our data. In the relevant literature few are the studies which assessed the efficacy of FDG investigation in patients with in-transit metastases. In a cohort of 64 patients - with a total of 80 cutaneous lesions - comparing the diagnostic possibilities of PET-CT with respect to whole- body MR, the first shows higher sensitivity if compared to whole-body magnetic resonance - 83% vs. 76% - but with a specificity of 65% [27]. Some Authors deem that coronal orientation of MR can be more difficult to interpret than axial plane if we are interested in detecting small subcutaneous metastases [27]. It is moreover highlighted the importance of anamnesis along with a thorough clinical examination [28,29], thus suggesting that further specific studies on in-transit metastases are yet needed.

The small population enrolled in this study didn’t permit a detailed statistical analyses and also the results obtained did not permit to achieve our primary goal; however we investigated a large number of lesions that allowed us to elaborate some considerations and to say that CT-PET and TT can integrate US information. HF-US is a time-consuming technique which implies the commitment of the operator, has relevant limitations in obese or non-collaborating patients and in those with lymphatic stasis. Moreover, differential diagnosis between still active metastatic disease and outcomes of anamnestic ECT-treated nodules can be quite demanding, in particular in the absence of color or power Doppler signals. It is furthermore impossible to exclude neoplastic residues inside the treated tissue. This aspect has not been considered in our study and such assumptions, which appear empirically logical, should be at any rate confirmed by further studies. Studies about the changes occurring in the morphological aspect of the US detectable metastatic lesions, after therapy, are not available: the only available data are relative to the reduction of post-perfusion vascularization [32,33]. Both agree in underlining, also in the relatively short post-operative period (about one week), a sharp reduction of vascularization, associated with high ultrasound specificity and sensitivity; such conclusions are in agreement with our data but the small dimensions of the lesions in our series envisage a low probability to detect a significant vascularization with doppler. In our opinion all the techniques we utilized has to be applicable early after treatment because of the transitory modifications induced by therapy. Furthermore, ultrasound has clear limits when applied to areas already treated with ECT, also after a rather long time from treatment, but similar problems can also be found with TT, since fibrotic tissue can interfere with infrared transmission thus making the area appear as “cold”. Similar phenomena are as well reported with TT in deep lesions, especially in obese patients and those with lymphatic stasis.

In conclusion, our preliminary results demonstrate that HF-US cannot at the moment be replaced by other available techniques in the diagnosis of in-transit metastases, especially for the small ones. However, PET-CT could have a real role in detecting deeper in transit metastases and TT could have a role in detecting large lesions and both could be useful in integrating the US results. Further studies in a larger patient population will be needed to confirm our results.

## Abbreviations

18-FDG: 18-fluoro-2-deoxy-D-glucose
ECT: Electrochemoterapy
HF-US: High frequency-ultrasound
PET-CT: Positron emission tomography-computed tomography
TT: Telethermography

## References

[1] Weide B, Faller C, Büttner P, Pflugfelder A, Leiter U, Eigentler TK, Bauer J, Forschner A, Meier F, Garbe C: **Prognostic factors of melanoma patients with satellite or in-transit metastasis at the time of stage III diagnosis.** *PLoS One* 2013, **8:**e6313. 10.1371/journal.pone.0063137 PMC3639278 23638183

[2] Chaudhary S, Bansal C: **Husain: Literature meta-analysis of zosteriform cutaneous metastases from melanoma and a clinico-histopathological report from India.** *Ecancermedicalscience* 2013, **7:**324. 10.3332/ecancer.2013.324 PMC3680231 23781279

[3] Maio M, Ascierto P, Testori A, Ridolfi R, Bajetta E, Queirolo P, Guida M, Romanini A, Chiarion-Sileni V, Pigozzo J, Di Giacomo AM, Calandriello M, Didoni G, van Baardwijk M, Konto C, Lucioni C: **The cost of unresectable stage III or stage IV melanoma in Italy.** *J Exp Clin Cancer Res* 2012, **31:**91. 10.1186/1756-9966-31-91 PMC3542007 23116062

[4] Savoia P, Fava P, Nardó T, Osella-Abate S, Quaglino P, Bernengo MG (2009). Skin metastases of malignant melanoma: a clinical and prognostic survey. Melanoma Res.

[5] Dummer R, Hauschild A, Guggenheim M, Keilholz U, Pentheroudakis G, ESMO Guidelines Working Group (2012). Cutaneous melanoma: ESMO Clinical Practice Guidelines for diagnosis, treatment and follow-up. Ann Oncol.

[6] Balch CM, Gershenwald JE, Soong SJ, Thompson JF, Atkins MB, Byrd DR, Buzaid AC, Cochran AJ, Coit DG, Ding S, Eggermont AM, Flaherty KT, Gimotty PA, Kirkwood JM, McMasters KM, Mihm MC, Morton DL, Ross MI, Sober AJ, Sondak VK (2009). Final version of 2009 AJCC melanoma staging and classification. J Clin Oncol.

[7] Solivetti FM, Elia F, Guerrisi A, Desiderio F, Santaguida M, Sperduti I, Cavallotti A, Di Carlo A: **Cutaneous melanoma follow-up: appropriateness of requests for ultrasound tests – the S.Gallicano National Referral Centre Experience.** *J Exp Clin Cancer Res* 2013, **32:**73. 10.1186/1756-9966-32-73 PMC3851827 24422765

[8] Ascierto PA, Botti G, Del Vecchio M (2013). Melanoma Guidelines.

[9] Marcoval J, Ferreres JR, Penín RM, Piulats JM, Caminal JM, Fabbra A (2011). Descriptive analysis of cutaneous recurrence patterns in patients with melanoma. Actas Dermosifiliogr.

[10] Clemente-Ruiz de Almiron A, Serrano-Ortega S (2012). Risk factors for in-transit metastasis in patients with cutaneous melanoma. Actas Dermosifiliogr.

[11] Spugnini EP, Citro G, Baldi A: **Adjuvant electrochemotherapy in veterinary patients: a model for the planning of future therapies in humans.** *J Exp Clin Cancer Res* 2009, **28:**114. 10.1186/1756-9966-28-114 PMC2739846 19682373

[12] Sersa G (2006). The state-of-the-art of electrochemotherapy before the ESOPE study: advantages and clinical uses. Eur J Cancer.

[13] Caracò C, Mozzillo N, Marone U, Simeone E, Benedetto L, Di Monta G, Di Cecilia ML, Botti G, Ascierto PA: **Long-lasting response to electrochemotherapy in melanoma patients with cutaneous metastasis.** *BMC Cancer* 2013, **13:**564. 10.1186/1471-2407-13-564 PMC4219480 24289268

[14] Turley RS, Raymond AK, Tyler DS (2011). Regional treatment strategies for in-transit melanoma metastasis. Surg Oncol Clin N Am.

[15] Testori A, Tosti G, Martinoli C, Spadola G, Cataldo F, Verrecchia F, Baldini F, Mosconi M, Soteldo J, Tedeschi I, Passoni C, Pari C, Di Pietro A, Ferrucci PF (2010). Electrochemotherapy for cutaneous and subcutaneous tumor lesions: a novel therapeutic approach. Dermatol Ther.

[16] Mandavan A, Rao Ravuri P, Konathan R (2013). High-resolution ultrasound imaging of cutaneous lesions. Indian J Radiol Imaging.

[17] Trimboli P, Giovannella L, Valabrega S, Andrioli M, Baldelli R, Cremonini N, Rossi F, Guidobaldi L, Barnabei A, Rota F, Paoloni A, Rizza L, Fattorini G, Latini M, Ventura C, Falasca P, Orlandi F, Crescenzi A, D’Ambrosio F, Cantisani V, Romanelli F, Negro R, Saggiorato E, Appetecchia M: **Ultrasound features of medullary thyroid carcinoma correlate with cancer aggressiveness: a retrospective multicenter study.** *J Exp Clin Cancer Res* 2014, **33:**87. 10.1186/s13046-014-0087-4 PMC4219042 25344474

[18] Solivetti FM, Di Luca Sidozzi A, Pirozzi G, Coscarella G, Brigida R, Eibenshutz L (2006). Sonographic evaluation of clinically occult in-transit and satellite metastases from cutaneous malignant melanoma. Radiol Med.

[19] Catalano O, Caracò C, Mozzillo N, Siani A (2010). Locoregional spread of cutaneous melanoma: sonography findings. Am J Roentgenol.

[20] Nazarian LN, Alexander AA, Kurtz AB, Capuzzi DM, Rawool NM, Gilbert KR, Mastrangelo MJ (1998). Superficial melanoma metastases: appearances on gray-scale and color Doppler sonography. Am J Roentgenol.

[21] Shada AL, Dengel LT, Petroni GR, Smolkin ME, Acton S, Slingluff CL (2013). Infrared thermography of cutaneous melanoma metastases. J Surg Res.

[22] Di Carlo A (1995). Thermography and the possibilities for its applications in clinical and experimental dermatology. Clin Dermatol.

[23] Poljak-Blazi M, Kolaric D, Jaganjac M, Zarcovic K, Skala K, Zarcovic N (2009). Specific thermographic changes during Walker 256 carcinoma development: differential infrared imaging of tumour, inflammation and haematoma. Cancer Detect Prev.

[24] Herman C, Cetingul MP (2011). Quantitative visualization and detection of skin cancer using dynamic thermal imaging. J Visual Exp.

[25] Santa Cruz GA, Berlotti J, Marìn J, Gonzales SJ, Gossio S, Alvarez D, Roth BM, Menendez P, Pereira MD, Albero M, Cubau L, Orellano P, Liberman SJ (2009). Dynamic infrared imaging of cutaneous melanoma and normal skin in patients treated with BNCT. Appl Radiat Isot.

[26] Solivetti FM, Elia F, Latini A, Cota C, Cordiali-Fei P, Di Carlo A: **AIDS-Kaposi sarcoma anc classic Kaposi sarcoma: are different ultrasound pattern related to different variants?** *J Exp Clin Cancer Res* 2011, **30:**40. 10.1186/1756-9966-30-40 PMC3095540 21489270

[27] Acland KM, O’Doherty MJ, Russell-Jones R (2000). The value of positron emission tomography scanning in the detection of subclinical metastatic melanoma. J Am Acad Dermatol.

[28] Friedman KP, Wahl RL (2004). Clinical use of positron emission tomography in the management of cutaneous melanoma. Semin Nucl Med.

[29] Laurent V, Traush G, Brout O, Olivier P, Felbinger J, Regent D (2010). Comparative study of two whole-body imaging techniques in the case of melanoma metastases: advantages of multi-contrast MRI examination including a diffusion-weighted sequence in comparison with PET-CT. Eur J Radiol.

[30] Pfannenberg C, Aschoff P, Schanz S, EschmannPlathow C, Eigentler TK, Garbe C, Brechtel K, Vonthein R, Bares R, Claussen CD, Schlemmer HP (2007). Prospective comparison of 18F-fluorodeoxyglucose positron emission tomography/computed tomography and whole-body magnetic resonance imaging in staging of advanced malignant melanoma. Eur J Cancer.

[31] Covarelli P, Burini G, Barberini F, Caracappa D, Boselli C, Nova G, Castellani E, Rulli A (2013). The Integrated role of ultrasonography in the diagnosis of soft tissue metastases from melanoma: preliminary report of a single-center experience and literature review. In Vivo.

[32] Lassau N, Chami L, Peronneau P (2007). Imaging of melanoma: accuracy of ultrasonography before and after contrast injection for diagnostic and early evaluation of treatments. Bull Cancer.

[33] Hochedez P, Lassau N, Bonvalot S, Bidault S, Leclère J, Avril MF (2003). Treatment of local recurrent melanomas by isolated limb perfusion: value of Doppler ultrasonography. J Radiol.

